# Associations between mental illness, TB risk and migrant status

**DOI:** 10.5588/ijtldopen.24.0260

**Published:** 2024-12-01

**Authors:** S.E. Hayward, K.L. Kristensen, A. Deal, J.H. Petersen, T. Lillebaek, S. Hargreaves, M. Norredam, J.S. Friedland

**Affiliations:** ^1^ Institute for Infection and Immunity, School of Health and Medical Sciences, City St George’s, University of London, London, UK;; ^2^ Faculty of Public Health and Policy, London School of Hygiene & Tropical Medicine, London, UK;; ^3^ International Reference Laboratory of Mycobacteriology, Statens Serum Institut, Copenhagen, Denmark;; ^4^ Department of Pulmonary and Infectious Diseases, Nordsjaellands Hospital, Hilleroed, Denmark;; ^5^ Section of Biostatistics, Department of Public Health, University of Copenhagen, Copenhagen, Denmark;; ^6^ Global Health Section, Department of Public Health, University of Copenhagen, Copenhagen, Denmark;; ^7^ Danish Research Centre for Migration, Ethnicity and Health, Department of Public Health, University of Copenhagen, Copenhagen, Denmark;; ^8^ Section for Immigrant Medicine, Department of Infectious Diseases, Hvidovre University Hospital, Hvidovre, Copenhagen, Denmark.

**Keywords:** tuberculosis, mental health, migrant, cohort, Europe

## Abstract

**BACKGROUND:**

TB and mental illnesses are public health priorities that often co-exist, with migrants in high-income countries being at risk for both conditions. This study investigates whether mental illness influences TB risk and examines the impact of migration status.

**METHODS:**

A nationwide prospective cohort study was conducted in Denmark from 1994–2015, involving migrants matched 1:6 to Danish-born individuals. Cox regression models, adjusted for age, sex and migrant status, were used to assess the effect of mental disorders on TB risk.

**RESULTS:**

Both migrants and non-migrants with mental disorders showed elevated TB incidence (*n* = 1,189,273). After adjusting for age and sex, the hazard ratio (HR) for TB in those with any mental disorder was 3.62 (95% CI 2.99–4.39, *P* < 0.001) compared to those without mental disorders. The effect was more substantial in Danish-born individuals (HR 15.51, 95% CI 12.05–19.95, *P* < 0.001) than in migrants (HR 1.37, 95% CI 0.99–1.90, *P* = 0.055). Sub-analyses highlighted a significant effect of substance use (HR 5.49, 95% CI 4.46–6.76, *P* < 0.001) and psychosis (HR 4.19, 95% CI 1.74–10.08, *P* = 0.001) and borderline significance for affective/anxiety/stress-related disorders (HR 1.64, 95% CI 0.98–2.73, *P* = 0.058) on TB risk.

**CONCLUSIONS:**

People with mental illnesses, particularly psychotic and substance use disorders, have increased TB incidence and represent a high-risk population for targeted screening and treatment. TB programmes should integrate holistic mental health care.

## RÉSUMÉ

**CONTEXTE:**

La TB et les troubles mentaux constituent des enjeux majeurs de santé publique qui se manifestent fréquemment de manière concomitante, les migrants originaires de pays à revenu élevé étant particulièrement vulnérables à ces deux pathologies. Cette recherche vise à déterminer si les troubles mentaux affectent le risque de TB et à analyser l'effet du statut migratoire sur cette relation.

**MÉTHODES:**

Une étude de cohorte prospective à l'échelle nationale a été menée au Danemark entre 1994 et 2015, impliquant des migrants qui ont été appariés dans un ratio de 1 :6 à des personnes nées au Danemark. Des modèles de régression de Cox, ajustés en fonction de l'âge, du sexe et du statut migratoire, ont été utilisés afin d'analyser l'impact des troubles mentaux sur le risque de TB.

**RÉSULTATS:**

Les migrants et les non-migrants présentant des troubles mentaux ont révélé une prévalence élevée de la TB (*n* = 1 189 273). Après ajustement des données en fonction de l'âge et du sexe, le rapport de risque (HR) pour la TB chez les personnes souffrant de troubles mentaux s’élevait à 3,62 (IC 95% 2,99–4,39; *P* < 0,001) par rapport à ceux ne présentant pas de tels troubles. Cet effet était particulièrement marqué chez les personnes nées au Danemark (HR 15,51 ; IC à 95% 12,05–19,95 ; *P* < 0,001) par rapport aux migrants (HR 1,37 ; IC à 95% 0,99–1,90 ; *P* = 0,055). Les sous-analyses ont révélé un impact significatif de la consommation de substances (HR 5,49 ; IC à 95% 4,46–6,76 ; *P* < 0,001) et de la psychose (HR 4,19 ; IC à 95% 1,74–10,08 ; *P* = 0,001), ainsi qu'une signification marginale pour les troubles affectifs/nerveux (HR 1,64 ; IC à 95% 0,98–2,73 ; *P* = 0,058) sur le risque de TB.

**CONCLUSIONS:**

Les personnes atteintes de troubles mentaux, notamment les troubles psychotiques et les troubles liés à la consommation de substances, présentent un risque accru de TB et constituent une population vulnérable nécessitant un dépistage et un traitement spécifiques. Il est essentiel que les programmes de lutte contre la TB incluent des soins de santé mentale complets.

Both TB and mental health are urgent global health priorities, with 1.3 million deaths from TB worldwide in 2022,^1^ and 5% of global disability-adjusted life-years (DALYs) attributable to mental disorders.^2^ TB and mental illnesses often co-exist, yet the nature of associations is not well-understood, with complex and bidirectional relationships between the two conditions.^3^ Chronic symptoms of TB, lengthy treatment courses, medication side effects, and stigma may all trigger or exacerbate mental illness in TB patients.^4^ There is increasing interest in whether, conversely, poor mental health may drive increased TB incidence, with a recent systematic review identifying a limited number of cohort studies―all based in Asia―showing that mental illness is associated with subsequent increased TB risk.^5–9^ In addition, there is robust evidence showing an association between alcohol and drug use and TB risk.^10–12^ These data are supported by evidence that psychosocial stress, mental illness and substance use directly influence the immune system and susceptibility to infection,^13^ including pathways relevant to *Mycobacterium tuberculosis* infection and progression to TB disease.^14,15^ However, the effect of mental illness on TB risk has not been comprehensively assessed in any European cohort.

In high-income countries in Europe, migrants make up a high proportion of TB cases: 33% in European Union/European Economic Area (EU/EEA) countries and over 70% in parts of Northern and Western Europe, including Nordic countries and the United Kingdom.^16^ Migrants in Denmark have a significantly higher TB incidence (120/100,000 person-years [py], 95% CI 115–126) than the Danish-born (4/100,000 py, 95% CI 3–4).^17^ This disparity closes with increased time since migration but remains high for over a decade.^18–20^ Across Europe, migrants, especially refugees, asylum seekers and undocumented migrants, are at increased risk of certain mental disorders.^21^ In Denmark, refugees have a higher overall risk of having a first-time psychiatric contact for a mental disorder than native Danes,^22^ but family reunification migrants have a lower risk.^23^ In addition, migrants in Denmark diagnosed with post-traumatic stress disorder (PTSD) and depression have higher rates of infectious disease than those without diagnosed psychiatric disorders (incidence rate ratio [IRR] 1.89, 95% CI 1.45–2.48; *P* < 0.01).^24^ However, the association between mental health and TB risk, and variations by migrant status, have not been fully explored to date in Europe or elsewhere.

Denmark has an excellent network of interlinkable, longitudinal, population-based registries.^25^ Few other European countries routinely collect or disaggregate disease data by migrant status.^26^ While previous work uses Danish registry data to look specifically at mental illness and substance abuse (MISA) in TB cases and controls through a case-control design, finding increased odds of TB among people with MISA than those without,^27^ we use a prospective cohort design to explore the association between all mental and behavioural disorders and subsequent TB risk. This is the first population-based cohort study in Europe to examine the association between mental health and TB risk and to explore variations between migrants (refugees/asylum seekers and family-reunified migrants) and non-migrants.

## METHODS

### Study population

**Figure 1.**
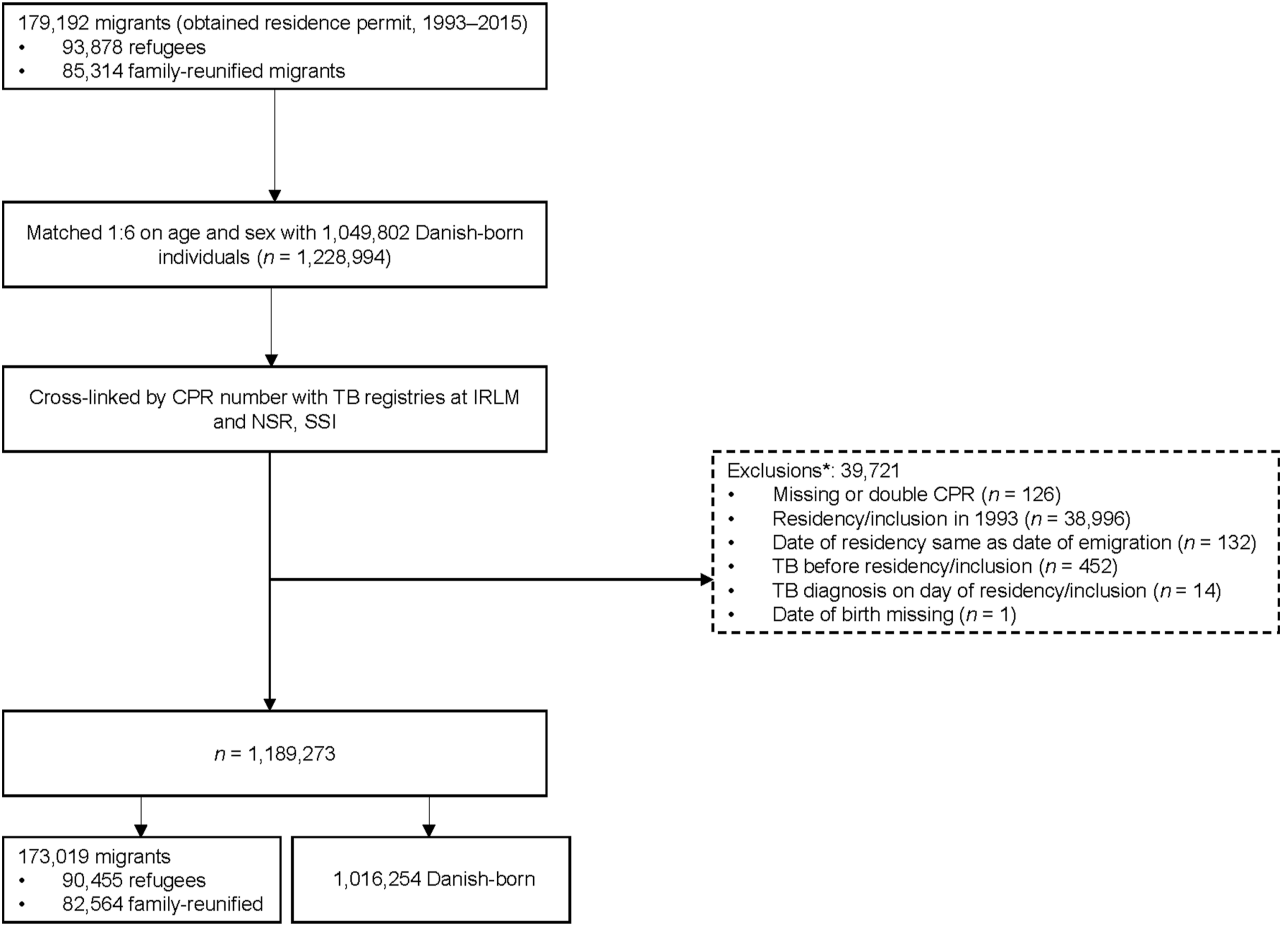
Flow chart of selection process for study cohort. *Where a migrant is excluded, their matched controls are also excluded. CPR = civil person registration; IRLM = International Reference Laboratory of Mycobacteriology; NSR = National Surveillance Register; SSI = Statens Serum Institut.

This prospective cohort study uses data from the Danish Migrant Cohort, a nationwide register-based cohort of all migrants (adult and child) who obtained a residence permit in Denmark between 1 January 1993 and 31 December 2015, identified through the Danish Immigration Service (Figure 1). Migrants were grouped according to their legal grounds of residence: 1) former asylum seekers and quota refugees (henceforth ‘refugees’), and 2) family-reunified migrants. In addition, a native Danish-born comparison group (born to Danish-born parents) was identified through Statistics Denmark, matched in a 1:6 ratio by age and sex. Migrants were included from the date of receiving residency; Danish-born controls from the date when their migrant match was granted residency. Individuals with a diagnosis of TB on or before this date were excluded from analyses. The cohort generation and matching process has been previously described.^17,28^ Reasons for exclusion related to this process are listed in Figure 1. Only migrants who received residency between 1 January 1994 and 31 December 2015 were included in this study, as International Classification of Disease, Tenth Revision (ICD-10) classifications were not in use until 1 January 1994.

### Data linkages

All Danish residents are assigned a civil person registration number (CPR) at birth or date of residency. The CPR can be used to track individuals through public registries. Data on age, sex, immigration, emigration and death were obtained from Statistics Denmark. Using each individual’s CPR, the cohort was linked to the International Reference Laboratory of Mycobacteriology (IRLM) and the Danish National Surveillance Register (NSR), both located at Statens Serum Institut (SSI), Copenhagen. All cases of active TB disease, both pulmonary and extrapulmonary, were included. Diagnosis was either microbiologically confirmed or based on clinical/paraclinical findings suggestive of TB, prompting TB treatment. Data on mental illness were obtained from the National Patient Registry (NPR), which includes records of any hospital visit (inpatient, outpatient and emergency room, henceforth ‘hospital contact’). Mental illness is defined based on a hospital contact involving an ICD-10 diagnosis of F00 through F99, which includes all mental and behavioural disorders (henceforth ‘mental disorders’). We defined sub-groups for substance use (F10–F19), psychotic (F20–F29), and affective and anxiety/stress-related (F30–F48) disorders.

### Statistical methods

Descriptive analyses were carried out using chi-squared and t-tests to describe baseline characteristics of the study cohort at inclusion (sex, age, region of origin, residency status, mental disorders, TB). In survival analyses, individuals were followed from the date of entry into the cohort until censoring due to one of the following events: 1) TB diagnosis, 2) emigration, 3) death, and 4) study end. Person-years were divided into two portions of follow-up: person-years after a hospital contact for a mental disorder (‘mental disorder’) and person-years without any prior contact (‘no mental disorder’). TB incidence rate was expressed as the number of newly diagnosed cases per 10,000 py, with IRRs calculated to compare mental disorder and no mental disorder groups. Cumulative incidence functions were plotted based on a marginal competing-risk regression model to compare how the incidence of TB in those with and without a mental disorder varies between migrants and Danish-born.

A multivariable Cox proportional hazards model was used to calculate the hazard ratio (HR), comparing those with and without a mental disorder, adjusting for age (at the start of the portion of follow-up, as a continuous variable) and sex. We decided a priori to evaluate effect modification by migrant status; as such, a new model was built including migrant status as an interaction term, with hazard ratios reported separately for migrants and Danish-born (and, in an additional model, for refugees and family-reunified migrants), also adjusting for age and sex as previously. The presence of effect modification was confirmed using a likelihood ratio test (LRT), which tested the difference between the model with and without the interaction term. Analyses were repeated for sub-groups of mental disorder (substance use, psychotic, affective/anxiety/stress-related) following the above process, redefining portions of follow-up as before/without and after the first hospital contact for the specific condition being evaluated (e.g. first substance use contact). In sensitivity analyses, analyses were repeated as above and stratified to define portions of follow-up according to the first hospital contact by setting (emergency room, inpatient, or outpatient). Results were considered statistically significant at *P* < 0.05, with *P* values for borderline significant results approaching *P* = 0.05 also reported. All analyses were carried out in StataSE v18 (Stata, College Station, TX, USA).

### Ethical approval

This study was approved by the Danish Data Protection Agency, Valby (2016-41-4576) and by the University of Copenhagen, Copenhagen, Denmark (514-0231/18-3000).

## RESULTS

### Baseline characteristics of the study cohort

Baseline characteristics of the study cohort (*n* = 1,189,273) are shown in Table 1 for both migrant and Danish-born groups. The mean age at inclusion was 28.6 years, with a slightly higher proportion of females (54.1%). There were no significant differences in age and sex between migrants and Danish-born due to the matching process. Migrants originated from diverse regions, including high TB burden areas across the Middle East and North Africa (28.0%), Eastern Europe and Central Asia (25.4%), South-East Asia (20.6%), and sub-Saharan Africa (13.9%). Refugees accounted for slightly more than half of the migrant cohort (52.3%).

**Table 1.** Baseline characteristics of study cohort.

	Migrants *n* (%)	Danish-born *n* (%)	*P* value*
Total, *n*	173,019	1,016,254	
Follow-up time, years, mean	10.90	12.30	
Sex			
Male	79,510 (46.0)	465,894 (45.8)	0.40
Female	93,509 (54.1)	550,360 (54.2)	
Age at inclusion, years, mean ± SD	28.57 ± 13.96	28.56 ± 13.27	0.87
Region of origin			
Eastern Europe and Central Asia	43,991 (25.4)		
Europe, North America and Oceania	14,850 (8.6)		
Latin America and the Caribbean	5,058 (2.9)		
Middle East and North Africa	48,426 (28.0)		
South-East Asia	35,602 (20.6)		
Sub-Saharan Africa	23,981 (13.9)		
Stateless	1,111 (0.6)		
Residency status			
Refugee	90,455 (52.3)		
Quota refugee	9,696 (5.6)		
Asylum seeker	80,759 (46.7)		
Family-reunified migrants	82,564 (47.7)		
To Danish/Nordic citizens	57,666 (33.3)		
To immigrants	9,852 (5.7)		
To refugees	15,046 (8.7)		
Mental disorders			
Any mental disorder	5,435 (3.1)	33,559 (3.3)	
Substance use	1,594 (27.7)	17,690 (48.7)	
Psychosis	231 (4.0)	1,146 (3.2)	
Affective/anxiety/stress-related disorder	2,826 (49.1)	10,447 (28.8)	
Other mental disorder	1,103 (19.2)	7,040 (19.4)	
No mental disorder	167,584 (96.9)	982,695 (96.7)	0.001^†^
TB status			
Any TB	1,933 (1.1)	411 (0.04)	<0.001^†^
Pulmonary	1,038 (55.3)	349 (87.9)	<0.001^†^
Extrapulmonary	838 (44.7)	48 (12.1)	
No TB	171,086 (98.9)	1,015,843 (100.0)	

* Pearson’s χ^2^ test used for categorical variables, *t*-test for continuous variables.

† Statistically significant.

SD = standard deviation.

Overall, 38,994 individuals (3.3%) had at least one hospital contact for a mental disorder during the follow-up period. Of these, 19,284 (49.5%) were related to substance use disorders, 1,377 (3.5%) to psychotic disorders, and 13,273 (34.0%) to affective or anxiety/stress-related disorders. Migrants had a higher proportion of affective/anxiety/stress-related disorders, while Danish-born individuals had a higher proportion of substance use disorders. There were 2,344 cases of TB during the study period, with the majority (82.5%) occurring in migrants. Migrants were significantly more likely to have TB disease than the Danish-born (χ^2^ = 8,700, *P* < 0.001) and had a significantly higher proportion of extrapulmonary TB than Danish-born individuals (χ^2^ = 146.21, *P* < 0.001).

### Increased TB incidence observed in both migrants and Danish-born with mental disorders

The incidence rate of TB was significantly higher in individuals with hospital contact for any mental disorder compared to those without (IRR 2.44, 95% CI 2.01–2.95, *P* < 0.001). Specifically, the TB incidence rate in those with a mental disorder was 3.87/10,000 py, while in those without a mental disorder, it was 1.58/10,000 py (Table 2).

**Table 2.** TB incidence by mental disorder status.

	Event (TB)	py*	Rate (95%CI)^†^	IRR (95%CI)	*P* value
No mental disorders	2,230	14.09	1.58 (1.52–1.65)	Reference	<0.001
Any mental disorder	114	0.29	3.87 (3.22–4.65)	2.44 (2.01–2.95)	

* Million py.

† Per 10,000 py.

py = person-years; CI = confidence interval; IRR = incidence rate ratio.

**Figure 2.**
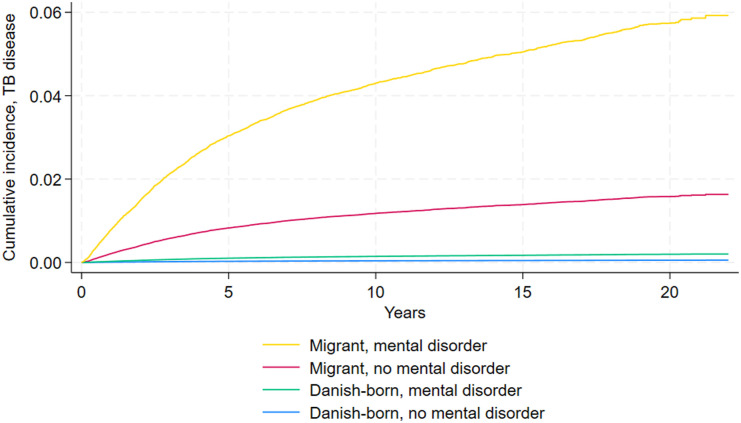
Cumulative incidence function of TB disease in study cohort.

Patients with a hospital contact for any mental disorder also had a higher cumulative incidence of TB than those without, in both migrants and Danish-born individuals (Figure 2). Additionally, migrants showed a higher overall cumulative incidence of TB compared to Danish-born individuals.

### Mental disorder associated with increased TB disease risk

Cox regression analyses revealed that hospital contact for any mental disorder was significantly associated with an increased subsequent risk of TB disease. After adjusting for age and sex, the hazard ratio (HR) for TB disease in individuals with any mental disorder was 3.62 (95% CI 2.99–4.39, *P* < 0.001) compared to those without a mental disorder (Table 3).

**Table 3.** Multivariable Cox regression analysis of factors associated with risk of TB.

	Total*		Migrants^†^		Danish-born^†^	
	HR (95%CI)	*P* value	HR (95%CI)	*P* value	HR (95%CI)	*P* value
Any mental disorder	3.62 (2.99–4.39)	<0.001	1.37 (0.99–1.90)	0.055	15.51 (12.05–19.95)	<0.001
Substance use	5.49 (4.46–6.76)	<0.001	2.92 (1.94–4.37)	<0.001	24.73 (19.12–31.98)	*<*0.001
Psychosis	4.19 (1.74–10.08)	0.001	1.55 (0.39–6.21)	NS	14.54 (4.67–45.30)	*<*0.001
Affective/anxiety/stress-related disorder	1.64 (0.98–2.73)	0.058	0.50 (0.24–1.05)	NS	5.49 (2.72–11.08)	*<*0.001

* Adjusting for age and sex.

† Adjusting for age and sex, allowing for interaction with migrant status.

HR = hazard ratio; CI = confidence interval; NS = not significant.

When evaluating effect modification by migrant status (LRT χ^2^ = 144.07, *P* < 0.001), the effect of mental disorder on TB risk was significantly stronger in Danish-born individuals (HR 15.51, 95% CI 12.05–19.95; *P* < 0.001) compared to migrants, in whom the effect approached borderline significance (HR 1.37, 95% CI 0.99–1.90; *P* = 0.055). Among migrants, the effect was more pronounced in refugees (HR 1.38, 95% CI 0.95–2.01; *P* = 0.09) than family-reunified migrants (HR 1.20, 95% CI 0.64–2.25; *P* = 0.56) (Supplementary Table S1).

In sensitivity analyses, significant associations between TB risk and mental disorder were observed across all hospital settings (emergency room, inpatient, outpatient). TB risk was highest in individuals with an emergency room contact for a mental disorder (HR 5.04, 95% CI 3.97–6.40), followed by inpatient (HR 4.25, 95% CI 3.27–5.52) and outpatient contacts (HR 3.64, 95% CI 2.67–4.95) (Supplementary Table S2). All results were statistically significant (*P* < 0.001).

### Substance use and psychosis associated with increased TB disease risk

Cox regression analyses by specific mental disorder categories showed that hospital contact for a substance use disorder (HR 5.49, 95% CI 4.46–6.76; *P* < 0.001) and a psychotic disorder (HR 4.19, 95% CI 1.74–10.08; *P* = 0.001) were significantly associated with increased TB risk. Affective or anxiety/stress-related disorders showed borderline significance (HR 1.64, 95% CI 0.98–2.73; *P* = 0.058) (Table 3).

For substance use disorders, the association with TB risk was significant in both migrants and Danish-born individuals (*P* < 0.001). However, the effect of psychotic and affective/anxiety/stress-related disorders was significant only in the Danish-born population (*P* < 0.001) and not in migrants (Table 3).

## DISCUSSION

Our data from a Danish nationwide cohort show that individuals with mental disorders, especially psychotic and substance use disorders, experience increased TB incidence. The association is particularly strong among the Danish-born, where mental disorders are associated with a much greater relative increase in TB risk (HR 15.51, 95% CI 12.05–19.95; *P* < 0.001) compared to migrants (HR 1.37, 95% CI 0.99–1.90; *P* = 0.055). Although the effect size is larger in Danish-born individuals, migrants with mental disorders represent the group with the highest absolute incidence of TB. This study adds to a small but growing body of evidence indicating that mental illness significantly increases the risk of subsequent TB disease, corroborating previous studies from Asia.

A study in South Korea (*n* = 64,744) reported an adjusted HR for TB incidence among those with depression compared to controls of 2.63 (95% CI 1.74–3.96; *P* < 0.001),^6^ while a study in Taiwan (*n* = 172,952) reported an adjusted HR of 1.15 (95% CI 1.03–1.28) for TB incidence among individuals with depression.^7^ Another study from Taiwan (*n* = 120,818) found an adjusted HR of 1.52 (95% CI 1.29–1.79; *P* < 0.001) for TB among those with schizophrenia,^8^ and a study from Nagasaki, Japan (*n* = 3,251) reported a relative risk of 3.04 (*P* < 0.005) for TB incidence among individuals with schizophrenia compared to population incidence rates.^9^

Our findings reveal an overall adjusted HR of 3.62 (95% CI 2.99–4.39; *P* < 0.001) for TB among individuals with any mental disorder compared to those without, which is significantly higher than the HRs observed in most previous studies. For substance use disorders, we report an HR of 5.49 (95% CI 4.46–6.76; *P* < 0.001), and for psychosis, an HR of 4.19 (95% CI 1.74–10.08; *P* = 0.001). Affective/anxiety/stress-related disorders were associated with a borderline significant increase in TB risk (HR 1.64, 95% CI 0.98–2.73; *P* = 0.058). These findings align with studies linking substance use disorders to TB,^10,12^ including a meta-analysis of four cohort studies that reported a pooled relative risk of 2.38 (95% CI 1.43–3.96) for TB among individuals with alcohol-related problems.^11^

This study is the first to use a prospective design to demonstrate that mental illness is associated with increased TB risk in a European population. Prior research using Danish national registry data has similarly found associations between mental disorders and infection risk, with PTSD being significantly associated with incident infections (HR 1.8, 95% CI 1.6–2.0).^29,30^ While the relative impact of mental disorder on TB risk is greatest in Danish-born individuals, the highest absolute TB incidence rates are observed among migrants with mental disorders, reflecting their high baseline TB incidence due to exposure in high-incidence countries and structural vulnerabilities post-migration.^31^

Our results indicate that refugees (HR 1.38, 95% CI 0.95–2.01; *P* = 0.09) have a higher TB risk associated with mental disorder compared to family-reunified migrants (HR 1.20, 95% CI 0.64–2.25; *P* = 0.56), though neither effect reaches statistical significance. The stronger association between mental disorder and TB risk among Danish-born individuals (HR 15.51, 95% CI 12.05–19.95; *P* < 0.001) reflects the fact that *M. tuberculosis* exposure is on average much lower among the native-born, and thus other risk factors, including mental illness, have a greater relative impact. In addition, Danish-born TB patients are more likely to experience social risk factors such as poverty, homelessness, and incarceration than migrant TB patients, potentially contributing to the observed association between mental illness and TB in this group.

In addition, there is a growing body of evidence that stress and mental illness can modulate immune function^13,32^ and infectious disease risk,^33^ including immune biomarkers relevant to TB,^15^ supporting a potential causal relationship as well. Schizophrenia, for example, has been linked to diverse immune alterations,^34^ while substance use can result in functional immune changes,^35–37^ increasing the risk of infectious diseases.^38^ Our sensitivity analyses support the hypothesis that mental illness severity influences TB risk, with emergency room contacts associated with the highest risk (HR 5.04, 95% CI 3.97–6.40), followed by inpatient (HR 4.25, 95% CI 3.27–5.52) and outpatient contacts (HR 3.64, 95% CI 2.67–4.95). This gradient suggests that individuals with more severe mental illness are at greater risk of developing TB, consistent with an earlier study showing a dose-response relationship between depression severity and TB risk.^6^

### Study strengths and limitations

This study has several strengths. It includes a large, population-based cohort (*n* = 1,189,273) followed for up to 22 years, using comprehensive Danish national registries to link TB and mental health data. This unique data set allows for a detailed analysis of the association between mental illness and TB risk in both migrants and Danish-born individuals. Our study is also the first in Europe to evaluate the effect of mental disorders on TB risk using a prospective design and to examine differences by migrant status, enabling a nuanced understanding of TB risk in these populations.

However, there are limitations to consider. Mental illness data were based on hospital contacts, meaning only severe cases requiring hospital care were captured. Less severe mental illness, treated in community settings, was not included. In addition, we were unable to adjust for socio-economic factors, such as income and education, which may confound the relationship between mental illness and TB. However, given the complexity of the interaction between socio-economic status, mental health, and TB, including such variables could introduce additional biases. Finally, certain migrant groups, such as undocumented migrants and labour migrants, were not captured in our cohort, potentially limiting the generalisability of the findings to these populations.^17,19,28^

### Public health implications

Despite these limitations, the findings of this study have important public health implications. Mental disorders, particularly substance use and psychosis, are associated with significantly increased TB risk. This highlights the need to target individuals with mental disorders for TB screening and treatment, especially in hospital settings. While TB screening programmes in Denmark and other low-incidence European countries tend to focus on migrants,^39–41^ our data show that Danish-born individuals with mental disorders represent a high-risk population for TB that could benefit from tailored public health interventions. Identifying people with mental disorders as a high-risk group for TB is crucial for developing targeted strategies that align mental health services with TB care, to improve both early TB detection and treatment adherence.^42–44^

## CONCLUSIONS

Our study demonstrates that mental disorders, particularly psychotic and substance use disorders, are associated with increased TB incidence in both migrants and Danish-born individuals in a low TB burden, European setting. Migrants with mental disorders experience the highest absolute TB incidence, while Danish-born individuals with mental disorders show the highest relative risk. These findings identify people with mental disorders as a high-risk group that should be targeted for TB screening and treatment. Integrated mental health and TB programmes could provide holistic care, improving outcomes for individuals with both conditions.

## Supplementary Material



## References

[1] World Health Organization. Global tuberculosis report, 2023. Geneva, Switzerland: WHO, 2023.

[2] Global, regional, and national burden of 12 mental disorders in 204 countries and territories, 1990–2019: a systematic analysis for the Global Burden of Disease Study 2019. Lancet Psychiatry. 2022;9(2):137–150. 35026139 10.1016/S2215-0366(21)00395-3 PMC8776563

[3] Sweetland AC, Addressing the tuberculosis–depression syndemic to end the tuberculosis epidemic. Int J Tuberc Lung Dis. 2017;21(8):852–861. 28786792 10.5588/ijtld.16.0584 PMC5759333

[4] Doherty AM, A review of the interplay between tuberculosis and mental health. Gen Hosp Psychiatry. 2013;35(4):398–406. 23660587 10.1016/j.genhosppsych.2013.03.018

[5] Hayward SE, The relationship between mental health and risk of active tuberculosis: a systematic review. BMJ Open. 2022;12(1):e048945. 10.1136/bmjopen-2021-048945 PMC8739435 34992103

[6] Oh KH, Depression and risk of tuberculosis: a nationwide population-based cohort study. Int J Tuberc Lung Dis. 2017;21(7):804–809. 28633706 10.5588/ijtld.17.0038

[7] Cheng KC, Increased risk of pulmonary tuberculosis in patients with depression: a cohort study in Taiwan. Front Psychiatry. 2017;8:235. 29180971 10.3389/fpsyt.2017.00235 PMC5694036

[8] Kuo SC, Incidence and outcome of newly-diagnosed tuberculosis in schizophrenics: a 12-year, nationwide, retrospective longitudinal study. BMC Infect Dis. 2013;13:351. 23895638 10.1186/1471-2334-13-351 PMC3729604

[9] Ohta Y, The epidemiological study of physical morbidity in schizophrenics–2. Association between schizophrenia and incidence of tuberculosis. Jpn J Psychiatry Neurol. 1988;42(1):41–47. 3260975 10.1111/j.1440-1819.1988.tb01954.x

[10] Lönnroth K, Alcohol use as a risk factor for tuberculosis: a systematic review. BMC Public Health. 2008;8:289. 18702821 10.1186/1471-2458-8-289 PMC2533327

[11] Imtiaz S, Alcohol consumption as a risk factor for tuberculosis: meta-analyses and burden of disease. Eur Respir J. 2017;50(1):1700216. 28705945 10.1183/13993003.00216-2017 PMC5540679

[12] Deiss RG, Tuberculosis and illicit drug use: review and update. Clin Infect Dis. 2009;48(1):72–82. 19046064 10.1086/594126 PMC3110742

[13] Glaser R, Stress-induced immune dysfunction: implications for health. Nat Rev Immunol. 2005;5(3):243–251. 15738954 10.1038/nri1571

[14] Zhang K, The interplay between depression and tuberculosis. J Leukoc Biol. 2019;106(3):749–757. 31254317 10.1002/JLB.MR0119-023R

[15] Hayward SE, A systematic review of the impact of psychosocial factors on immunity: implications for enhancing BCG response against tuberculosis. SSM Popul Health. 2020;10:100522. 31909166 10.1016/j.ssmph.2019.100522 PMC6939020

[16] European Centre for Disease Prevention and Control. Tuberculosis surveillance and monitoring in Europe 2022: 2020 data. Stockholm, Sweden: ECDC, 2022.

[17] Kristensen KL, Tuberculosis incidence among migrants according to migrant status: a cohort study, Denmark, 1993 to 2015. Euro Surveill. 2019;24(44):1900238. 31690363 10.2807/1560-7917.ES.2019.24.44.1900238 PMC6836680

[18] Norredam M, Duration of residence and disease occurrence among refugees and family reunited immigrants: test of the ‘healthy migrant effect’ hypothesis. Trop Med Int Health. 2014;19(8):958–967. 24889930 10.1111/tmi.12340

[19] Kristensen KL, Long-term risk of tuberculosis among migrants according to migrant status: a cohort study. Int J Epidemiol. 2020;49(3):776–785. 32380550 10.1093/ije/dyaa063

[20] Lillebaek T, Persistent high incidence of tuberculosis in immigrants in a low-incidence country. Emerg Infect Dis. 2002;8(7):679–684. 12095434 10.3201/eid0807.010482 PMC2730343

[21] WHO Regional Office for Europe. Mental health promotion and mental health care in refugees and migrants (Technical guidance on refugee and migrant health). Copenhagen, Denmark: WHO, 2018.

[22] Norredam M, Risk of mental disorders in refugees and native Danes: a register-based retrospective cohort study. Soc Psychiatry Psychiatr Epidemiol. 2009;44(12):1023–1029. 19294322 10.1007/s00127-009-0024-6

[23] Norredam M, Risk of mental disorders in family reunification migrants and native Danes: a register-based historically prospective cohort study. Int J Public Health. 2010;55(5):413–419. 20589410 10.1007/s00038-010-0162-3

[24] Lolk M, Somatic comorbidity among migrants with post-traumatic stress disorder and depression: a prospective cohort study. BMC Psychiatry. 2016;16(1):447. 27964720 10.1186/s12888-016-1149-2 PMC5153678

[25] Schmidt M, The Danish National Patient Registry: a review of content, data quality, and research potential. Clin Epidemiol. 2015;7:449–490. 26604824 10.2147/CLEP.S91125 PMC4655913

[26] European Centre for Disease Prevention and Control. Assessing the burden of key infectious diseases affecting migrant populations in the EU/EEA. Stockholm: ECDC; 2014.

[27] Nordholm AC, Mental illness, substance abuse, and tuberculosis risk. J Infect. 2023;86(5):e137. 10.1016/j.jinf.2023.01.035 36716977

[28] Norredam M. Migration and health: exploring the role of migrant status through register-based studies. Dan Med J. 2015;62(4):B5068. 25872539

[29] Momen NC, Association between mental disorders and subsequent medical conditions. N Engl J Med. 2020;382(18):1721–1731. 32348643 10.1056/NEJMoa1915784 PMC7261506

[30] Jiang T, Post-traumatic stress disorder and incident infections: a nationwide cohort study. Epidemiology. 2019;30(6):911–917. 31584893 10.1097/EDE.0000000000001071 PMC6779341

[31] Hayward S, Factors influencing the higher incidence of tuberculosis among migrants and ethnic minorities in the UK [version 2; referees: 2 approved]. F1000Res. 2018;7:461. 30210785 10.12688/f1000research.14476.1 PMC6107974

[32] Haapakoski R, Cumulative meta-analysis of interleukins 6 and 1β, tumour necrosis factor α and C-reactive protein in patients with major depressive disorder. Brain Behav Immun. 2015;49:206–215. 26065825 10.1016/j.bbi.2015.06.001 PMC4566946

[33] Andersson NW, Depression and the risk of severe infections: prospective analyses on a nationwide representative sample. Int J Epidemiol. 2015;45(1):131–139. 26708840 10.1093/ije/dyv333

[34] Ermakov EA, Immune system abnormalities in schizophrenia: an integrative view and translational perspectives. Front Psychiatry. 2022;13:880568. 35546942 10.3389/fpsyt.2022.880568 PMC9082498

[35] Xu E, Inflammasome in drug abuse. Int J Physiol Pathophysiol Pharmacol. 2017;9(6):165–177. 29348793 PMC5770513

[36] Adams C, Alcohol use disorder and circulating cytokines: a systematic review and meta-analysis. Brain Behav Immun. 2020;89:501–512. 32805393 10.1016/j.bbi.2020.08.002

[37] Pasala S, Impact of alcohol abuse on the adaptive immune system. Alcohol Res. 2015;37(2):185–197. 26695744 10.35946/arcr.v37.2.04 PMC4590616

[38] Sarkar D, Alcohol and the immune system. Alcohol Res. 2015;37(2):153–155.

[39] Dara M, Tuberculosis care among refugees arriving in Europe: a ERS/WHO Europe Region survey of current practices. Eur Respir J. 2016;48(3):808–817. 27492827 10.1183/13993003.00840-2016

[40] Margineanu I, Country-specific approaches to latent tuberculosis screening targeting migrants in EU/EEA countries: a survey of national experts, September 2019 to February 2020. Euro Surveill. 2022;27(12):2002070. 35332865 10.2807/1560-7917.ES.2022.27.12.2002070 PMC8950856

[41] Langholz Kristensen K, Tuberculosis screening among newly arrived asylum seekers in Denmark. Infect Dis (Lond). 2022;54(11):819–827. 36000199 10.1080/23744235.2022.2106380

[42] Lee G, Impact of mental disorders on active TB treatment outcomes: a systematic review and meta-analysis. Int J Tuberc Lung Dis. 2020;24(12):1279–1284. 33317672 10.5588/ijtld.20.0458 PMC7740071

[43] Sweetland AC, Tuberculosis: an opportunity to integrate mental health services in primary care in low-resource settings. Lancet Psychiatry. 2018;5(12):952–954. 30241700 10.1016/S2215-0366(18)30347-X PMC6489124

[44] Sweetland AC, Integrating tuberculosis and mental health services: global receptivity of national tuberculosis program directors. Int J Tuberc Lung Dis. 2019;23(5):600–605. 31097069 10.5588/ijtld.18.0530 PMC6558966

