# Supplementary material for: Associations between mental illness, TB risk and migrant status
Source: IJTLD Open. 2024 Dec 1;1(12):564–70. doi: 10.5588/ijtldopen.24.0260 (PMC11636499; doi:10.5588/ijtldopen.24.0260)
Supplement: Supplementary file 1 [file ijtldopen24-0260_supplementarydata1.docx]

# Associations between mental illness, TB risk and migrant status

**SUPPLEMENTARY DATA**

Supplementary Table S1. Multivariable Cox regression analysis of factors associated with risk of TB, stratified by migrant type.

|  | **Refugee*** | | **Family-reunified migrant*** | |
| --- | --- | --- | --- | --- |
|  | **HR (95% CI)** | ***p* value** | **HR (95% CI)** | ***p* value** |
| **Any mental disorder** | 1.38 (0.95-2.01) | *p=*0.09 | 1.20 (0.64-2.25) | *p*=0.56 |

*Adjusting for age and sex, allowing for interaction with migrant type

TB=tuberculosis, HR=hazard ratio, CI=confidence interval

Supplementary Table S2. Multivariable Cox regression analysis of factors associated with risk of TB, stratified by hospital setting.

| **Any mental disorder** | **Total*** | |
| --- | --- | --- |
|  | **HR (95% CI)** | ***p* value** |
| **Emergency room** | 5.04 (3.97-6.40) | *p*<0.001 |
| **Inpatient** | 4.25 (3.27-5.52) | *p*<0.001 |
| **Outpatient** | 3.64 (2.67-4.95) | *p*<0.001 |

*Adjusting for age and sex

TB=tuberculosis, HR=hazard ratio, CI=confidence interval
